# MEMS-metasurface–enabled mode-switchable vortex lasers

**DOI:** 10.1126/sciadv.adq6299

**Published:** 2024-11-20

**Authors:** Chuanshuo Wang, Chao Meng, Xianglong Mei, Lili Gui, Paul C. V. Thrane, Hao Chen, Fei Ding, Kun Xu, Sergey I. Bozhevolnyi

**Affiliations:** ^1^ State Key Laboratory of Information Photonics and Optical Communications, Beijing University of Posts and Telecommunications, Beijing 100876, China.; ^2^ Centre for Nano Optics, University of Southern Denmark, Campusvej 55, Odense M DK-5230, Denmark.; ^3^ SINTEF Smart Sensors and Microsystems, Gaustadalleen 23C, 0737 Oslo, Norway.

## Abstract

Compared to conventional lasers limited to generating static modes, mode-switchable lasers equipped with adjustable optics significantly enhance the flexibility and versatility of coherent light sources. However, most current approaches to achieving mode-switchable lasers depend on conventional, i.e., inherently bulky and slow, optical components. Here, we demonstrate fiber lasers empowered by electrically actuated intracavity microelectromechanical system (MEMS)–based optical metasurface (MEMS-OMS) enabling mode switching between fundamental Gaussian and vortex modes at ~1030 nm. By finely adjusting the voltage applied to the MEMS mirror, high-contrast switching between Gaussian (*l* = 0) and vortex (*l* = 1, 2, 3, and 5, depending on the OMS arrangement) laser modes is achieved, featuring high mode purities (>95%) and fast responses (~100 microseconds). The proposed intracavity MEMS-OMS–enabled laser configuration provides an at-source solution for generating high-purity fast-switchable laser modes, with potential applications ranging from advanced optical imaging to optical tweezers, optical machining, and intelligent photonics.

## Teaser

Dynamic lasers with MEMS-tunable metasurfaces are achieved for efficient, fast switching between Gaussian and vortex modes.

## INTRODUCTION

Over the past decade, optical configurations enabling flexible light field manipulation have witnessed rapid development, garnering considerable attention for the direct modulation and emission of structured beams from laser cavities (*1*–*4*). Of particular interest are laser systems for generating vortex beams that carry orbital angular momentum (OAM), defined by a spatially varying spiral phase exp(*il*φ), where φ is the azimuthal angle around the optical axis and *l* represents the topological charge, indicating the number of wavefront twists per unit wavelength. Vortex beams are in great demand for applications in optical telecommunications (*5*, *6*), stimulated emission depletion microscopy (*7*, *8*), laser interferometry (*9*–*11*), OAM holography (*12*–*15*), spiral phase contrast imaging (*16*–*18*), particle manipulation (*19*–*21*), and optical machining (*22*–*24*). The transition from spatial Gaussian or fiber linearly polarized LP_01_ modes to OAM modes is typically achieved by incorporating beam-shaping optics such as diffraction gratings (*25*), vortex wave plates (VWPs) (*26*, *27*), q-plates (*28*), and mode selective couplers (*29*, *30*) into the cavity. This integration not only enhances the generation efficiency and beam stability but also contributes to the realization of compact and user-friendly structured light sources (*1*, *2*). Very recently developed integrated optical vortex microcombs (*31*, *32*) use meticulously designed angular grating-dressed nonlinear microring resonators to control both the OAM and frequency of the generated microcomb lasers, representing a substantial advancement toward on-chip high-dimensional structured light sources.

Optical metasurfaces (OMSs), emerged as revolutionary planar (artificial) metamaterials, offer distinct advantages of compact sizes, flexible light field manipulation, and ease of integration. OMSs have gradually evolved into becoming a crucial omnipresent tool for intricate light field operations (*33*–*36*), contributing significantly to the miniaturization and functional expansion of light sources (*37*, *38*). Exceptional performances in OMS-assisted vortex lasers with static mode emission have been demonstrated through the integration of the plasmonic OMSs (*39*–*41*). Notably, our previous research introduced a platform using gap-surface plasmon OMSs, capable of generating wavelength-tunable and high-purity OAM beams from linear (*40*) and figure-9 (*41*) cavity fiber lasers. Nevertheless, these implementations were limited in their functionalities by relying on static intracavity OMSs with well-defined optical responses set during the design and fabrication.

Dynamic laser systems with switchable OAM orders overcome the limitations of lasers generating static modes by offering laser sources with greatly enhanced flexibility and versatility. Traditional passive adjustment schemes in laser systems involve the combination of beam-shaping [like OMSs (*42*, *43*), q-plates (*44*), and VWPs (*45*)] and polarization optics, such as quarter-wave plates (QWPs), inside the cavity. Switching between two opposite OAM orders can be achieved by switching the incident spin states (by rotating polarization optics) on the beam-shaping optics (*42*–*45*). However, the latter is inherently slow, requiring careful synchronization of multiple intracavity components and thereby intricate cavity configuration. Sroor *et al*. (*46*) proposed a rotatable J-plate OMS, with different vortex phase profiles encoded for orthogonal polarization directions, thereby capable of switching between two different OAM orders. This approach greatly facilitated miniaturization and simplified the cavity complexity, although still suffering from limited switching speeds. Commercial spatial light modulators (SLMs) allow for real-time flexible digital control of laser modes by altering the loaded phase holograms with approximately millisecond switching speed (*47*–*49*). Recently developed SLMs based on ferroelectric liquid crystal (*50*) and lithium niobate (*51*) can achieve faster switching, with response times at the level of hundreds of microseconds and nanoseconds, respectively. However, these technologies now exhibit limitations in phase tuning ranges and/or considerable large pixel sizes (see table S1). Moreover, conventional liquid crystal SLMs exhibit low damage thresholds, rendering them susceptible to damage under high power, particularly when used within an intracavity framework. In all-fiber lasers based on an acousto-optic mode converter (AOMC), rapid switching (~0.23 ms) between ±1-order OAM beams was achieved by adjusting the frequency of the radio waves applied to the AOMC (*52*, *53*). However, the mode conversion occurring in few-mode fibers, where OAM modes are generated through the superposition of multiple degenerate modes, presents considerable challenges in generating high-order OAM beams.

**Fig. 1.**
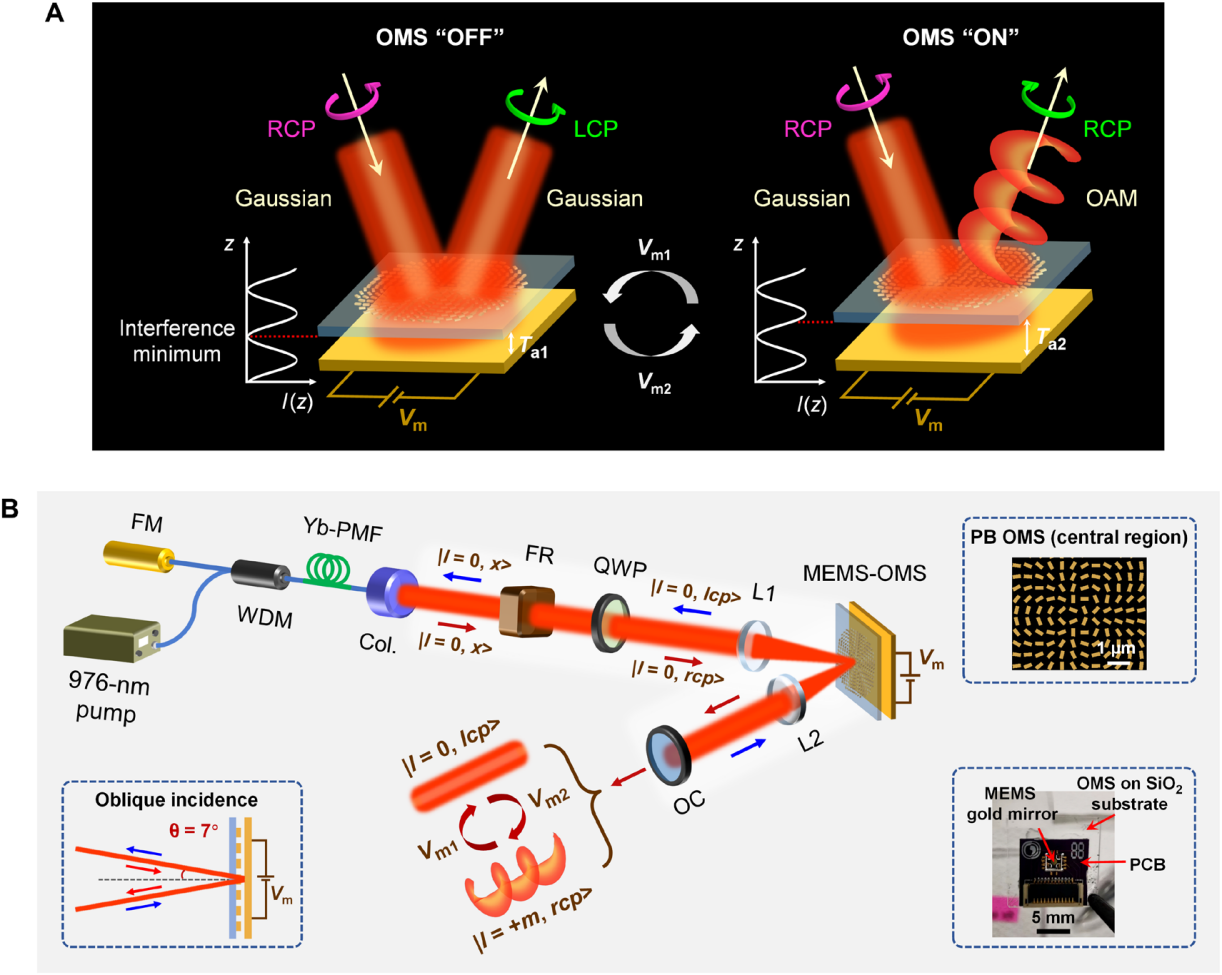
Mode switchable vortex laser design. (**A**) Working principle of the MEMS-OMS component for reconfigurable switching between Gaussian (|*l* = 0, *lcp*>) and OAM (|*l* = +*m*, *rcp*>, *m* is a nonzero integer) modes, under Gaussian beam incidence (|*l* = 0, *rcp*>). (**B**) Schematic of the MEMS-OMS–enabled V-shaped mode-switchable vortex fiber laser. FM, fiber mirror; WDM, wavelength division multiplexer; Yb-PMF, ytterbium-doped polarization-maintaining fiber; Col., collimator; FR, Faraday rotator; QWP, quarter-wave plate; L1, lens 1 (*f*_1_ = 75 mm); L2, lens 2 (*f*_2_ = 50 mm); OC, output coupler (R:T = 90:10). The top right inset depicts the OMS design (*l* = 5) with Pancharatnam-Berry (PB) phase encoded. The bottom left inset shows the 7° oblique incident/reflected beam to/from the MEMS-OMS in the practical laser cavity construction. The bottom right inset is a typical photo of the MEMS-OMS component, consisting of a PB OMS fabricated on a SiO_2_ substrate, a thin-film MEMS gold mirror, and a printed circuit board (PCB) for electrical connections.

Here, capitalizing on our developed piezoelectric microelectromechanical system (MEMS)–based dynamic OMSs (*54*–*58*), we propose and demonstrate intracavity MEMS-OMS–empowered fiber lasers capable of high-contrast switching between Gaussian and vortex laser modes at ~1030 nm. The MEMS-OMS dynamic response is achieved by OMS positioning at two different locations within the interference pattern created by the incident beam and reflected beam from the movable MEMS mirror (Fig. 1A). The polarization and mode evolution inside the laser cavity achieve a self-consistent solution, warranting efficient and stable laser operation (Fig. 1B and fig. S1). This work establishes an approach that uses ultracompact dynamic MEMS-OMSs for intracavity generation of structured coherent light beams with reconfigurable mode properties, demonstrating thereby a previously unidentified paradigm in tunable laser source design.

## RESULTS

### Working principle of MEMS-OMS–enabled mode-switchable vortex lasers

The dynamic MEMS-OMS component in our laser cavity comprises a Pancharatnam-Berry (PB) OMS fabricated on a SiO_2_ substrate, a bottom MEMS mirror, and a printed circuit board (PCB) for electrical connections (Fig. 1B, bottom right inset). The air gap *T*_a_ between the OMS and the MEMS mirror can be accurately controlled by the actuation voltages *V*_m_. The MEMS-OMS operates in the periodic Fabry-Pérot (FP) region with negligible near-field coupling between the MEMS mirror and the OMS (*55*, *56*). The overall optical response of the MEMS-OMS is determined by the paired phase singularities in the [*T*_a_, λ] parameter space, resulting from the interplay of plasmonic and FP resonances (fig. S2) (*55*, *59*). From an intuitive perspective, when an actuating voltage *V*_m1_ is applied to the MEMS mirror, the OMS layer can be precisely located at the interference minimum of the incident and reflected fields (separated from the MEMS mirror with *T*_a1_ = ~*k* × λ/2, *k* is nonnegative integers) and enters the “OFF” state (Fig. 1A and fig. S3). Thereby, the optical response of the MEMS-OMS is independent of the meta-atom dimensions (fig. S4). The entire component then exhibits mirror-like operation. Specifically, when a Gaussian beam with right-hand circular polarization (RCP) |*l* = 0, *rcp*> is incident upon it, the reflected beam exhibits left-hand circular polarization (LCP): |*l* = 0, *lcp*>. For another case, when the MEMS mirror is actuated with a specific voltage *V*_m2_, the OMS layer is positioned away from the interference minimum [separated from the MEMS mirror with *T*_a2_ and not necessarily located at the maxima of *I*(*z*)]. The OMS switches to the “ON” state, and the entire MEMS-OMS operates as an efficient VWP. In addition to the polarization handedness inversion induced by reflection, the light reflected from MEMS-OMS also undergoes the combined effects of the encoded PB phase profile (Fig. 1B, top right inset) and half-wave plate (HWP) function. Thus, the polarization state of the reflected beam remains unchanged, manifesting as the RCP state with nonzero OAM order: |*l* = +*m*, *rcp*> (*m* is a nonzero integer). This transformation from Gaussian and OAM modes also applies to LCP incident light and results in an OAM mode with opposite topological charge |*l* = −*m*, *lcp*> due to the spin-dependent nature of PB phase.

**Fig. 2.**
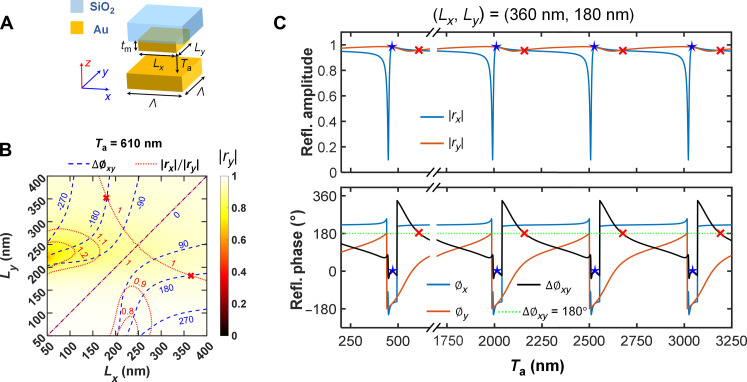
MEMS-OMS unit cell design. (**A**) Schematic of the MEMS-OMS unit cell. The thickness of the gold (Au) nanobrick and the unit cell period are fixed: *t*_m_ = 50 nm and Λ = 450 nm. The side lengths *L_x_* and *L_y_* of the nanobrick are variables. The air gap *T*_a_ between the nanobricks and the MEMS mirror can be adjusted by applying voltage to, and thus moving, the MEMS mirror. (**B**) Complex reflection coefficient *r* calculated as a function of nanobrick dimensions *L_x_* and *L_y_* at the wavelength of λ = 1030 nm for *T*_a_ = 610 nm. The color maps represent the reflection amplitude for normally incident *y*-polarized light. The blue and red contour lines indicate the reflection phase difference Δϕ*_xy_* and the ratio of reflection amplitudes |*r_x_*|/|*r_y_*| between *x*- and *y*-polarized incidence, respectively. (**C**) Evolution of the reflection amplitude |*r*_*x*(*y*)_*|*, phase ϕ_*x*(*y*)_, and phase difference Δϕ*_xy_* as a function of the air gap *T*_a_ for selected MEMS-OMS unit cell (*L_x_*, *L_y_*) = (360 nm, 180 nm). The red cross and blue star markers represent the ON (VWP operation) and OFF (mirror-like operation) states of the OMS at different air gap *T*_a_ within different FP orders (*k* = 1, 4, 5, and 6), respectively.

Our MEMS-OMS–integrated fiber laser, as illustrated in Fig. 1B, enables fast switching between Gaussian and OAM modes. The V-shaped laser cavity is formed between a fiber mirror (FM), a reflective MEMS-OMS, and a partially transparent output coupler (OC). The fiber components are connected by polarization-maintaining fibers. A 976-nm laser is coupled into the cavity with a wavelength division multiplexer, serving as the pump for the laser system. The ytterbium-doped polarization-maintaining fiber absorbs the injected pump power and provides gain to amplify the light circulating in the laser cavity. Initially, an *x*-polarized Gaussian beam |*l* = 0, *x*>, with a beam waist diameter of 2 mm, is coupled from the collimator. The Faraday rotator (FR) rotates the polarization direction of the transmitted beam by 45° in a clockwise fashion (regardless of the incident direction) to ensure that the polarization state of the intracavity beam is self-consistent during each round trip. The MEMS-OMS at *T*_a2_ is designed as an HWP (Fig. 2) and encoded with PB phase in the cross-polarized channel under circular-polarized light incidence. A QWP is placed in front of the MEMS-OMS to convert the intracavity light between linear (|*l* = 0, 45°>) and circular polarization states (|*l* = 0, *rcp*>). The generated RCP Gaussian beam, denoted as |*l* = 0, *rcp*>, is obliquely incident at a 7° angle (Fig. 1B, bottom left inset) and weakly focused onto the MEMS-OMS using a lens (L1, *f* = 75 mm). By varying the voltage applied to the MEMS mirror, rapid switching of the reflected beam between LCP Gaussian |*l* = 0, *lcp*> and RCP vortex modes |*l* = +*m*, *rcp*> can be achieved. The generated beams are collected by another lens (L2, *f* = 50 mm) positioned along the optical axis of the reflected light and then directly coupled out of the cavity through a partially transparent OC [reflectance:transmittance (R:T) = 90:10]. The majority of the intracavity laser beam is reflected by the OC and remains inside the cavity for efficient optical feedback, thus ensuring stable laser operation. Following the re-modulation of the MEMS-OMS, the beams regulated by MEMS-OMS at different operation states can be restored to Gaussian beams while maintaining the same circular polarization state and then return to their original state |*l* = 0, *x*> following the joint polarization conversion through QWP and FR. Subsequently, they are coupled back into the optical fiber through the fiber collimator. The intracavity beam undergoes multiple reflections and evolutions between the FM and the OC, facilitating self-consistent polarization and mode distribution at each position within the cavity (fig. S1). Note that the intracavity dynamic MEMS-OMS not only switches between different laser modes but also maintains self-consistent mode evolution within the laser cavity, which is essential for efficient and stable laser operation.

### MEMS-OMS design

The MEMS-OMS unit cell consists of a movable bottom MEMS gold (Au) mirror and an Au meta-atom fabricated on a SiO_2_ substrate (Fig. 2A). The operation wavelength of the OMS and the thickness of the nanobricks are specified as λ = 1030 nm and *t*_m_ = 50 nm, respectively. Meanwhile, we set the unit cell size of Λ = 450 nm to avoid higher-order diffraction and excitation of surface waves. To minimize the loss introduced by the MEMS-OMS within the laser cavity, achieving an efficient mode transformation is essential at the VWP operation state. Following the rule of an ideal HWP, viz., equal reflection amplitudes (|*r_x_*| = |*r_y_*| = 1) and a π phase difference (Δϕ*_xy_* = ϕ*_x_* − ϕ*_y_* = 180°) along the *x* and *y* directions, we calculated the complex reflection coefficients as a function of the nanobrick sizes (*L_x_*, *L_y_*) with different air gap sizes *T*_a_ under normally incident linearly polarized light (fig. S5; see the “Numerical simulations” section). By tracking the intersection points (red cross markers) between the contour lines of equal reflection amplitudes |*r_x_*|/|*r_y_*| and π phase difference Δϕ*_xy_* and considering the limitations of aspect ratio (~1) in nanofabrication, the optimized MEMS-OMS with HWP function is (*L_x_*, *L_y_*) = (360 nm, 180 nm) at *T*_a_ = 610 nm (Fig. 2B). Meanwhile, this MEMS-OMS unit cell features high reflection amplitudes (~0.96) for both *x*- and *y*-polarized incidence.

The optical response of the MEMS-OMS is tunable by moving the MEMS mirror and thereby changing the MEMS-OMS separation between the mirror and OMS. Figure 2C depicts the evolution of the reflection amplitudes |*r*_*x*(*y*)_|, phase ϕ_*x*(*y*)_, and phase difference Δϕ*_xy_* as a function of the air gap *T*_a_ for designed unit cell. At *T*_a_ = 610 nm + (*k* − 1) × λ/2 (*k* = 1, 2, 3, …), the OMS layer is at the ON state. The phase difference Δϕ*_xy_* remains 180°, and high reflection amplitudes |*r*_*x*(*y*)_| ~ 1 persist near these air gaps (red cross markers), adhering to the ideal HWP. For mirror-like operation, i.e., *T*_a_ = 465 nm + (*k* − 1) × λ/2 (*k* = 1, 2, 3, …), the OMS layer is at the OFF state. The reflection amplitudes |*r*_*x*(*y*)_| are ~1, while the phase difference Δϕ*_xy_* is 0° (blue stars). The OMS ON and OFF states follow a periodic manner (with a period of λ/2), owing to its FP nature (*55*, *56*) (fig. S2; see the “Numerical simulations” section).

**Fig. 3.**
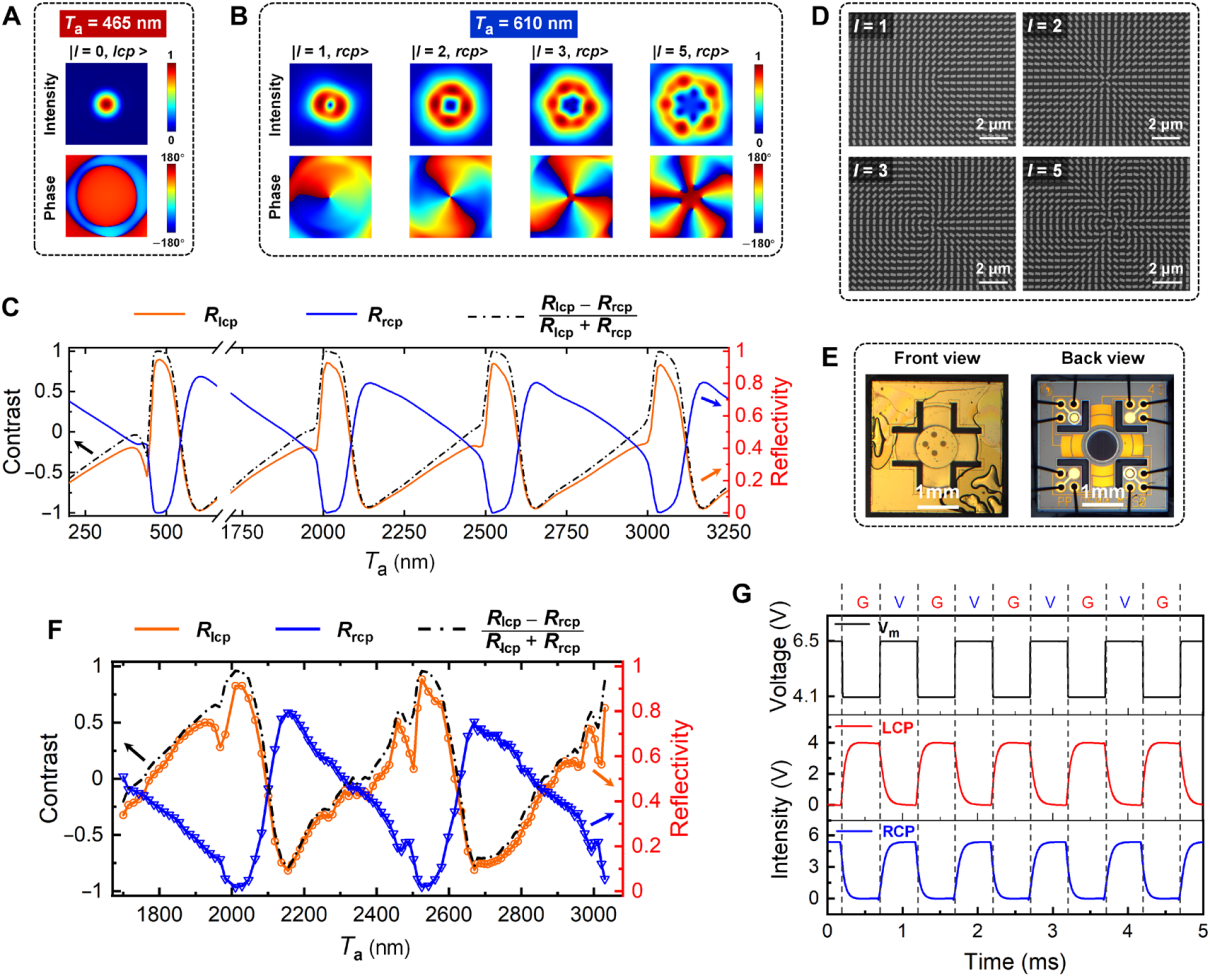
MEMS-OMS design, fabrication, and characterization (λ = 1030 nm). (**A**) Simulated intensity (first row) and phase profiles (second row) of the reflected Gaussian mode (|*l* = 0, *lcp*>) at *T*_a_ = 465 nm (*k* = 1), under RCP Gaussian beam (|*l* = 0, *rcp*>) incidence. (**B**) Simulated intensity (first row) and phase profiles (second row) of the reflected vortex modes (|*l*= +*m*, *rcp*>) with different topological charges (*l* = 1, 2, 3, and 5) at *T*_a_ = 610 nm (*k* = 1), under RCP Gaussian beam (|*l* = 0, *rcp*>) incidence. (**C**) Simulated reflectivity at LCP/RCP channels and corresponding polarization contrast (*l* = 1 MEMS-OMS) as a function of the air gap *T*_a_, under RCP Gaussian beam incidence. (**D**) Scanning electron microscopy images of the fabricated OMSs encoded with different topological charges (*l* = 1, 2, 3, and 5). (**E**) Optical microscope images of the assembled MEMS-OMS component, showing the piezoelectric material lead zirconate titanate (PZT) cantilevers holding a circular mirror above which four circular OMSs can be seen. (**F**) Measured reflectivity of the Gaussian and vortex modes at respective orthogonal LCP/RCP channels as a function of *T*_a_ (*l* = 1 MEMS-OMS). (**G**) Time response of the Gaussian (G) and vortex (V) beam switching measured at orthogonal LCP/RCP channels, with the MEMS mirror actuated with a periodic rectangle signal.

Based on the designed MEMS-OMS unit cell, the MEMS-OMS arrangements for dynamic switching between Gaussian and vortex beams with different topological charges can be realized (Fig. 3, A and B, and fig. S6). The meta-atom orientation in each MEMS-OMS unit cell is adjusted according to the PB phase required for generating vortex beams with *l* = 1, 2, 3, and 5. With a normally incident RCP Gaussian beam (|*l* = 0, *rcp*>), the reflected beam maintains a Gaussian beam (|*l* = 0, *lcp*>) for *T*_a_ = 465 nm, while it converts to a vortex beam (|*l* = +*m*, *rcp*>) for *T*_a_ = 610 nm. In addition, MEMS-OMS operating with larger air gap sizes, i.e., higher FP orders (*k* = 4 and 5), is also investigated, featuring well-preserved capability of high-contrast Gaussian/vortex mode switching (figs. S7 and S8). The reflected power from the MEMS-OMS is redistributed between the RCP and LCP channels, i.e., the Gaussian and vortex modes in a periodic manner, with air gaps *T*_a_ varying from 200 to 3250 nm (Fig. 3C, *l* = 1 MEMS-OMS). For the first FP order (*k* = 1), the efficiencies reach 95 and 85%, and the contrasts between the orthogonal polarization channels (*R*_lcp_ − *R*_rcp_)/(*R*_lcp_ + *R*_rcp_) are 0.99 and −0.97 at respective mirror-like and VWP operation states. Notably, the designed MEMS-OMS maintains its capability of high-efficiency and high-contrast mode switching with increased air gap sizes. As shown in Fig. 3C, at VWP operation states, the reflectivity only slightly drops from 85% at *T*_a_ = 610 nm (*k* = 1) to 80% at *T*_a_ = 3185 nm (*k* = 6), while the polarization contrast slightly changes from −0.97 to −0.93.

### MEMS-OMS fabrication and characterization

Using electron-beam lithography (EBL) and liftoff processes (see the “MEMS-OMS fabrication and assembly” section), four OMSs were fabricated on a single glass substrate for dynamic generation of vortex beams with different topological charges of *l* = 1, 2, 3, and 5, respectively. Each OMS was fabricated with a circular aperture of 100 μm diameter to align with a slightly focused beam incident onto the MEMS-OMS in the later laser cavity construction. The scanning electron microscopy images illustrate the central area of each PB OMS arrangement for generating different OAM orders (Fig. 3D). The fabricated OMS is carefully aligned and assembled with a gold-coated 1-mm-diameter MEMS mirror (Fig. 3E, front view). For the assembly of the MEMS mirror and OMS, it is crucial to ensure the absence of any particles or irregularities from both sides, which could potentially hinder the movement of the MEMS mirror and affect the parallelism between the two surfaces. The mirror is wire bonded to eight electrodes on the PCB (Fig. 3E, back view). By applying voltages to these electrodes, the MEMS-OMS separation can be adjusted at a nanometer-level precision. The MEMS-OMS separation *T*_a_ can be experimentally estimated using white light interferometry, characterized with an initial *T*_a_ = ~1.7 μm, and moving range of ~1.3 μm by applying voltages from 0 to 23 V (fig. S9; see the “Optical characterization” section).

Before implementing into the laser cavity, the MEMS-OMS performances are characterized with a homemade optical setup capable of polarization-resolved direct and Fourier plane imaging (see the “Optical characterization” section). Figure 3F presents the measured efficiency of the MEMS-OMS at λ = 1030 nm, with air gaps *T*_a_ varying from 1700 to 3100 nm (*l* = 1 MEMS-OMS). At the Gaussian operation state, where the OMS is at the OFF state and the MEMS-OMS functions essentially as a mirror, the component maintains a high reflectivity of 94% and a high polarization contrast of 0.96 between RCP and LCP channels. While at the VWP operation state, the conversion efficiency of the MEMS-OMS slightly decreases from 80% at *T*_a_ = 2155 nm (*k* = 4) to 76% at *T*_a_ = 2670 nm (*k* = 5), with the polarization contrast changes from −0.8 to −0.77. Although the efficiency is lower than those estimated in simulation, this is anticipated from the fabrication defects and increased loss due to practical polycrystalline gold and the thin titanium adhesive layer between the gold nanobricks and the SiO_2_ substrate. Despite fabrication imperfection, MEMS-OMS can effectively switch the reflected beams between Gaussian and vortex modes at respective circular polarization channels, captured using a complementary metal-oxide semiconductor (CMOS) camera (movie S1). The reflected beam from the MEMS-OMS was documented while incrementally increasing the actuating voltages, showcasing power redistribution between orthogonal circular polarization channels, as well as the periodic manner in the MEMS-OMS operation (fig. S10 and movie S2). As illustrated in Fig. 3G, the rise/fall times for the mirror and VWP operations, estimated at ~95/130 and 126/98 μs, respectively, were determined by actuating the MEMS mirror with periodic rectangular signals and detecting the reflected power at corresponding circular polarization channels using a photodetector (see the “Optical characterization” section). Note that the response time of the MEMS-OMS component is related to the intrinsic oscillation frequency of the MEMS mirror, which is dependent on design parameters such as geometry, weight, and stiffness (*60*). By optimizing the MEMS mirror for faster responses, an operation bandwidth in the megahertz range can be expected (*61*–*63*).

### Intracavity MEMS-OMS–enabled mode-switchable vortex lasers

**Fig. 4.**
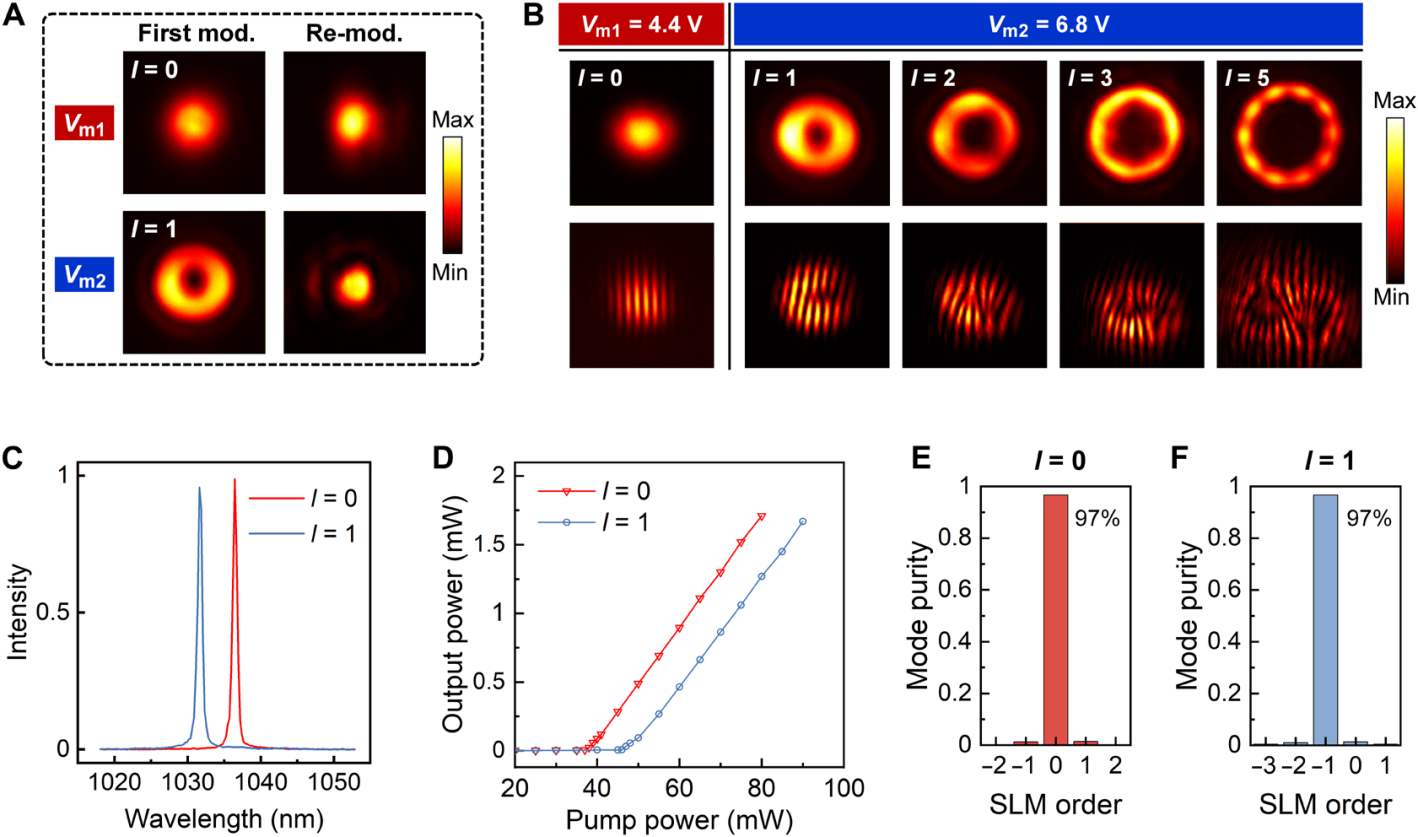
MEMS-OMS–enabled mode-switchable vortex lasers: experiment. (**A**) Self-consistent verification of the beam envelopes: measured intensity profiles of the first modulated and subsequent re-modulated beam by the MEMS-OMS at mirror-like (*V*_m1_ = 4.4 V, *k* = 4) and VWP operation (*V*_m2_ = 6.8 V, *k* = 4) states outside the cavity. (**B**) Measured intensity distributions (first row) and self-interference patterns (second row) of Gaussian (*l* = 0) and vortex beams (*l* = 1, 2, 3, and 5) emitted from the laser cavity. (**C**) Optical spectra of the mode-switchable laser at Gaussian (*l* = 0) and vortex (*l* = 1) mode operation states. (**D**) Measured output power of mode-switchable laser at Gaussian (*l* = 0) and vortex (*l* = 1) mode operation states as a function of pump power. (**E** and **F**) Mode purities of the emitted laser beams evaluated by an SLM.

The laser cavity integrated with the intracavity MEMS-OMS is illustrated in Fig. 1B. Note that, due to the slightly oblique incidence (7°) on the MEMS-OMS in the practical laser cavity, the optimal air gap (*T*_a_) for both mirror and VWP operations experiences a redshift by 10 nm (fig. S11; see the “Numerical simulations” section). The intracavity circulating laser beam satisfies a self-consistent condition, exhibiting identical polarization and transverse mode profiles for both forward and backward propagating waves at each position, as illustrated in fig. S1. Specifically, the feedback beams, which are reflected from the OC and interacting with the MEMS-OMS twice, have been experimentally examined, demonstrating a successful conversion back to a Gaussian-like beam without encoding any OAM order (Fig. 4A and fig. S12).

By varying the actuating voltage *V*_m_ applied to the MEMS mirror, controllable switching of the direct laser emission between Gaussian and vortex modes is realized. For example, with *V*_m1_ = 4.4 V (*k* = 4) and *V*_m2_ = 6.8 V (*k* = 4), the MEMS-OMS can be reconfigured between mirror-like and VWP operation states, resulting in switchable output laser between Gaussian and vortex modes. As shown in Fig. 4B, the resulting Gaussian and vortex laser beams exhibit distinct intensity profiles with central brightness and darkness, respectively. Slight nonuniformity in the vortex intensity profiles obtained can be attributed to the discretization of phase profiles at the MEMS-OMS design stage (fig. S13) and potential MEMS-OMS misalignment with the circulating beam in the laser cavity (fig. S14). Both effects can be mitigated by increasing the overall size of the MEMS-OMS in future developments (*64*). Furthermore, the phase profiles of the emitting laser beams are also verified through a self-interference method (*65*) (fig. S15; see the “Optical characterization” section), confirming that the high-quality MEMS-OMS enables dynamic laser mode switching. Note that the modulated beams (by MEMS-OMS) are mostly (90%) reflected back into the cavity by the OC to facilitate the laser oscillation. As a final comment, the vortex laser beams with different OAM orders (*l* = 1, 2, 3, and 5) are achieved through different OMS arrangements (Fig. 4B). For higher-order vortex lasers, the laser threshold increases because of the augmented loss incurred in the Gaussian-to-vortex and vortex-back-to-Gaussian conversion process (fig. S12).

The operation wavelength of the fiber laser is determined by the total gain and loss within the cavity. As illustrated in Fig. 4C, the central wavelength stabilizes at 1036 nm when the laser operates with Gaussian mode (*l* = 0) emission. In this case, the MEMS-OMS essentially functions as a mirror, making the lasing wavelength predominantly dependent on the other optical components inside the cavity. However, for vortex laser mode (*l* = +*m*) emission, the MEMS-OMS not only influences the polarization and mode profiles in part of the laser cavity but also introduces a wavelength-dependent loss owing to the non-perfect polarization/mode conversion capability. This influences the overall losses of the laser cavity, resulting in a slight shift of the central wavelength to 1031 nm. This slightly increased loss is also visualized by studying the laser threshold and the input-output curve of the proposed mode-switchable laser integrated with *l* = 1 MEMS-OMS (Fig. 4D). The laser, when emitting Gaussian beam, has a threshold pump power of 39 mW and a slope efficiency of 4.1%, while, in the vortex beam operation states, threshold pump power slightly rises to 47 mW with a slope efficiency of 4%. The integration of MEMS-OMS for higher-order vortex laser operation features larger insertion losses, evident from the increased threshold pump power (fig. S16). For instance, to achieve the vortex laser operation with *l* = 5, a threshold pump power of ~90 mW was required. The mode purity is characterized by loading spiral phase profiles corresponding to different OAM orders onto an SLM and demodulating the emitted beam from the laser cavity (fig. S17; see the “Optical characterization” section) (*66*). Taking *l* = 1 MEMS-OMS as an example, both Gaussian and vortex beams exhibit a high mode purity of 97% (Fig. 4, E and F). Similarly, the mode purity for higher-order OAM beams (*l* = 2, 3, and 5) consistently exceeds 95% (fig. S18). Furthermore, fig. S19 presents the comparison of the emitted laser intensity profiles at orthogonal polarization channels (LCP/RCP) under different MEMS-OMS operation states, showcasing high-contrast mode switching, consistent with the expectations from the simulation (Fig. 3C and figs. S6 to S8). The high-contrast, dynamic switching between Gaussian- and vortex-mode operations of the laser was visualized by applying a 1-Hz periodic rectangle signal to the MEMS mirror, documented in movie S3.

## DISCUSSION

In summary, we have demonstrated a MEMS-OMS–enabled dynamic laser capable of switching between Gaussian and vortex modes at around 1030 nm. The laser cavity, engineered in a V-shaped configuration and comprising an FM, a MEMS-OMS, and an OC, offers a straightforward and easily adjustable setup. By precisely adjusting the driving voltage of the MEMS mirror, switchable laser modes are generated with high purity (>95%) and fast switching speed (~100 μs). The collaboration between the intracavity MEMS-OMS, the FR, and the QWP ensures the self-consistent evolution of the polarization and mode distribution within the laser cavity for each roundtrip, across both Gaussian and vortex operation states. The switching between Gaussian and higher-order vortex beams (*l* = 2, 3, and 5) is also demonstrated, featuring high-contrast laser mode switching. Our proposed MEMS-OMS component and laser system show promise across several advanced applications. The dual–mode-switching capability of our laser configuration can be found beneficial for laser interferometry, offering unique advantages for detailed surface topology profiling and rotational measurements with enhanced sensitivity and accuracy (*9*–*11*). This capability is also well suited for encoding and retrieving information in advanced mode-multiplexed holography (*12*–*15*), enabling high-capacity data storage and dynamic displays. Furthermore, the developed MEMS-OMS component can be used in reconfigurable spiral phase contrast imaging (*16*–*18*), allowing real-time capture of both bright-field and edge-detected images. In particle manipulation, this fast–mode-switching capability facilitates precise and synchronous control of particle trapping, movement, and rotation (*19*–*21*). In optical machining, the continuously tunable mode profiles of our system allow real-time adaption to fabrication requirements involving different materials, shapes, geometries, and other parameters (*22*–*24*). Overall, our work represents a step forward in the development of dynamically controlled laser sources, with potential applications in advanced imaging, optical tweezers, optical machining, and other intelligent/adaptive photonic systems.

## MATERIALS AND METHODS

### Numerical simulations

The simulations of the MEMS-OMS unit cells were performed using COMSOL Multiphysics 5.6 with the Wave Optics module. First, the model was established for the SiO_2_/gold nanobrick/air configuration without the bottom MEMS gold mirror. The periodic boundary conditions are applied in both *x* and *y* directions to simulate a two-dimensional infinite array. The corners of the nanobrick are rounded with a 5 nm radius. The refractive index of gold is interpolated from the experimental data of Johnson and Christy (*67*). The refractive index of air is set as 1. The SiO_2_ layer is considered as a lossless dielectric material with refractive index of 1.46. The complex reflection and transmission coefficients are calculated as a function of nanobrick dimensions (*L_x_* and *L_y_*) with linearly *x*-/*y*-polarized light incidence from both directions (i.e., normal incidence from SiO_2_ or air). Then, the total reflection coefficient *r*_tot_ with a bottom gold mirror that is separated from the OMS with an air gap size of *T*_a_ can be calculated with FP equation

$$r_{\text{tot}}=r_{12}+\frac{t_{12}t_{21}r_{23}e^{i2kn_{2}T_{a}}}{1-r_{21}r_{23}e^{i2kn_{2}T_{a}}}$$

where *r_ij_* and *t_ij_* denotes the reflection and transmission coefficients, with the light incident on material *j* from material *i*. Each layer is numbered 1, 2, and 3, which refer to SiO_2_ substrate, air, and gold mirror, respectively, and **k** is the wave number in vacuum. The single OMS layer is located between layer 1 and 2, i.e., SiO_2_ and air, and its reflection/transmission coefficients *r*_12_, *r*_21_, *t*_12_, and *t*_21_ are calculated by COMSOL simulation. The reflection coefficient *r*_23_ of the gold mirror is calculated using the Fresnel equation.

Simulation for the oblique incidence was done for the designed MEMS-OMS unit cell (i.e., whole SiO_2_/gold nanobrick/air/gold mirror structure) using COMSOL Multiphysics 5.6. In simulation, the incident angle of excitation port was set to 7°, and the complex reflection coefficients of the designed MEMS-OMS unit cell as a function of air gap sizes was analyzed (fig. S11), featuring a redshift of optimized *T*_a_ by ~10 nm for achieving optimal mirror and VWP operation states.

The performances of different MEMS-OMSs (*l* = 1, 2, 3, and 5) are evaluated using FDTD Lumerical Solutions. Perfectly matched layers are used in the *x*, *y*, and *z* boundaries to truncate the simulation domain. Owing to computational demand, the OMS array in simulation is scaled down to 9 μm diameter, corresponding to 20 × 20 unit cells. Far-field intensity and phase distributions of the reflected beam from the MEMS-OMS are projected to orthogonal circular polarization channels.

### MEMS-OMS fabrication and assembly

The four OMSs (for *l* = 1, 2, 3, and 5 MEMS-OMS), each with 100 μm diameter, were fabricated using EBL, thin-film deposition, and liftoff techniques. First, a 100-nm poly(methyl methacrylate) A2 layer and a 40-nm conductive polymer layer were successively spin coated on a 14 mm–by–14 mm SiO_2_ substrate. The OMS patterns were defined on the substrate using EBL and then developed in 1:3 solution of methyl isobutyl ketone and isopropyl alcohol. Subsequently, a 2-nm titanium adhesion layer and a 50-nm gold layer were deposited using thermal evaporation. Lastly, the OMS layer was formed after a liftoff process in acetone.

The MEMS mirror was fabricated using standard semiconductor manufacturing processes. First, a bottom electrode layer of Pt, a thin-film layer of the piezoelectric material lead zirconate titanate (PZT), and gold top electrodes were successively deposited on a silicon-on-insulator wafer. Afterward, an annular trench was etched from the backside of the wafer with deep reactive ion etching, resulting in a central silicon mass suspended by a PZT membrane cantilevers. Then, the backside of the wafer was covered with 100-nm gold layer to make a MEMS mirror. By applying voltages to the top electrodes, the PZT membrane can be deformed, thus allowing the gold mirror in the center to be moved upward or downward. Last, the MEMS chip is glued to the SiO_2_ substrate with fabricated OMSs and, subsequently, glued to a PCB. Gold wire bonding between the MEMS mirror and PCB is applied for electrical connection. Typically, the initial *T*_a_ separation of the MEMS-OMS component is ~2 μm, when no voltage is applied, being determined by the size and viscosity of the glue used in the assembly process. The MEMS mirror has a total moving range of ~±1.2 μm with applied voltage ranges of 0 to 23 V to the inner/outer electrodes, which allows for the MEMS-OMS operating across several adjacent FP orders. The air gap sizes as a function of the applied voltages are estimated by monitoring the reflection spectrum from the unstructured area of a MEMS-OMS component using a white light source, as shown in fig. S9. Typically, the metasurface layer and the MEMS mirror can be maintained in substantial parallel alignment after assembly, with minor non-parallelism adjusted by tilting the MEMS mirror using piezoelectric electrodes.

### Optical characterization

The separation between the MEMS mirror and the OMS was precisely controlled using a power supply capable of applying 0- to 23-V voltage to the MEMS mirror. To estimate the air gap *T*_a_ between the MEMS mirror and the OMS, we experimentally obtained the broadband reflection spectrum from the nonstructured area of the MEMS-OMS component and fitted with a standard FP etalon model. As shown in fig. S9, a broadband white light source (halogen lamp) was directly through a beam splitter (BS) and focused onto the sample using a 5× objective. The reflected light was collected by the same objective and passed through a BS and a tube lens, generating the first image plane where an iris (ID1) was placed for filtering out the reflected light within the area slightly apart from the OMS structures. The first image was then transformed by a lens (L1, *f*_1_ = 150 mm) to the corresponding Fourier image, where another iris (ID2) was placed for extracting central area in the Fourier plane. After passing through lens 2 (L2, *f*_2_ = 125 mm), a real image is captured using a CMOS camera. Incorporating an additional flip lens (FL; *f*_FL_ =100 mm) allows for the acquisition of a Fourier plane image. Subsequently, a spectrometer is used to capture the reflection spectrum.

To characterize the switching speed of the MEMS-OMS between mirror-like and VWP operation states, we used a signal generator to actuate the MEMS mirror with a 1-kHz periodic rectangle signal. A QWP and a polarizing beamsplitter (PBS) were used to convert the Gaussian and vortex beams into linearly polarized beams and separated them into two orthogonal polarization channels. The reflected and transmitted beams from the PBS were collected by the photodetectors connected to an oscilloscope for visualizing and recording the corresponding modulated optical signals (Fig. 3G).

To study the phase profiles (i.e., topological charges) of the emitted laser beams, we constructed a Michelson interferometer (fig. S15). A BS (R:T = 50:50) splits the emitted beam from the laser cavity into two separate optical paths. The two separated beams are then reflected individually by two mirrors (M1 and M2), combined again with the same BS, and, lastly, collected by a CMOS camera.

To evaluate the OAM mode purity, we used a reflective SLM loaded with different spiral phases to demodulate the output laser beams and then recorded the intensity profiles of the demodulated beams by a CMOS camera. OAM mode purity can then be estimated by the ratio of the relative central intensities of the demodulated beams to that of the output beam (*66*). The CMOS contains 1280 × 1024 pixels, and the typical efficiency of the SLM is 67%. The CMOS was set to a fixed exposure time and first recorded a background intensity noise image to eliminate the influence of the surrounding environment. The central bright spot of the CMOS can be clearly observed when the loaded phase has the opposite order to that of the vortex beam from the laser (fig. S17). After subtracting the background noise from images recorded at different SLM orders, the 4 pixels in the center of each image were taken to calculate the average intensity, which is lastly normalized to obtain the power weight of each SLM order.
