# Supplementary material for: MEMS-metasurface–enabled mode-switchable vortex lasers
Source: Sci Adv. 2024 Nov 20;10(47):eadq6299. doi: 10.1126/sciadv.adq6299 (PMC11578166; doi:10.1126/sciadv.adq6299)
Supplement: Supplementary file 1 — Table S1 Figs. S1 to S19 Legends for movies S1 to S3 [file sciadv.adq6299_sm.pdf]

Science Advances
AAAS

Supplementary Materials for

MEMS-metasurface–enabled mode-switchable vortex lasers

Chuanshuo Wang *et al.*

Corresponding author: Chao Meng, chao@mci.sdu.dk; Lili Gui, liligui@bupt.edu.cn; Kun Xu, xukun@bupt.edu.cn; Sergey I. Bozhevolnyi, seib@mci.sdu.dk

Sci. Adv. **10**, eadq6299 (2024)
DOI: 10.1126/sciadv.adq6299

The PDF file includes:

Table S1
Figs. S1 to S19
Legends for movies S1 to S3

Other Supplementary Material for this manuscript includes the following:

Movies S1 to S3

Table S1. Comparison of key characteristics estimated for different SLMs and MEMS-OMS.

Ref.	Model name	Device type	Response time	Efficiency	Pixel size	Note
47	LCOS-SLM X110468E	Liquid crystal on silicon SLM	ms level	86%	20 μm	–
48	Texas DLP3000 DMD	Digital micro-mirror device SLM	0.25 ms	1.5%	7.5 μm	–
50	Lab work	Ferroelectric Liquid crystal SLM	250 μs	77%	3.94 μm	Limited phase tuning range of 1.9π
51	Lab work	lithium niobate SLM	200 ns	–	425 μm	Limited to 1D wavefront control
This work	Lab work	MEMS-OMS	100 μs	> 80%	450 nm	–

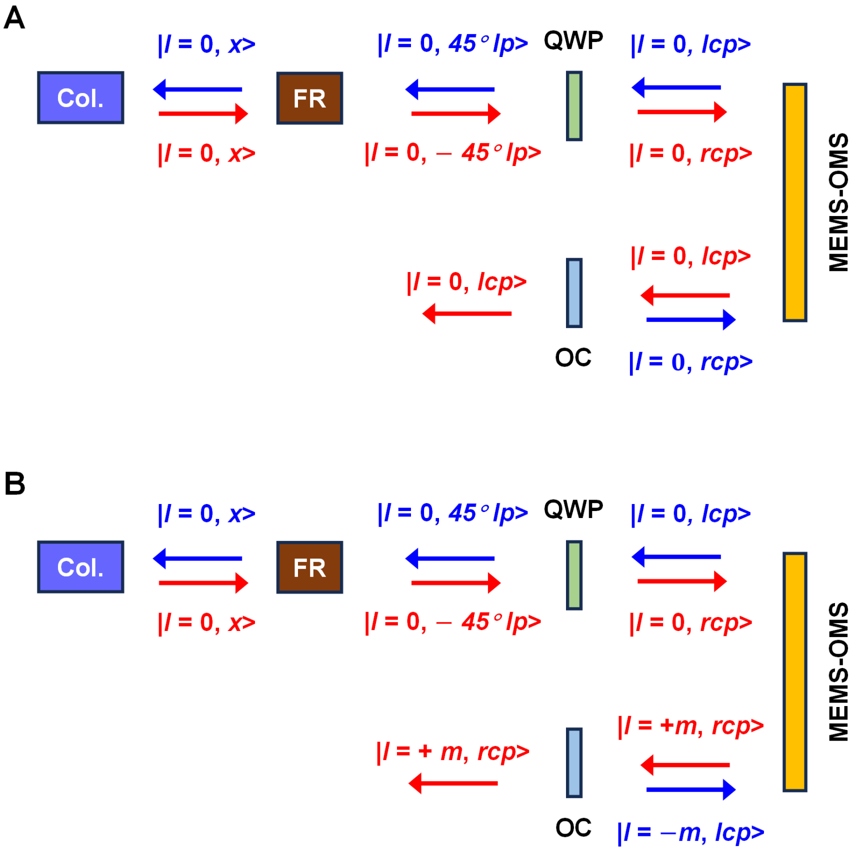

Fig. S1. Intracavity beam state evolution for (A) Gaussian (l = 0) and (B) vortex modes (l = + m) emission. (**A**) When the MEMS-OMS is at mirror-like operation state, the intracavity beam along the light path maintains the Gaussian mode and only exhibits the polarization handedness reversal due to reflection. (**B**) When the MEMS-OMS is at VWP operation state, in addition to the polarization handedness reversal induced by reflection, the beam also undergoes spatial mode conversion. The reflected vortex beam from the output coupler (OC) undergoes a simultaneous polarization handedness inversion and topological charge flipping.

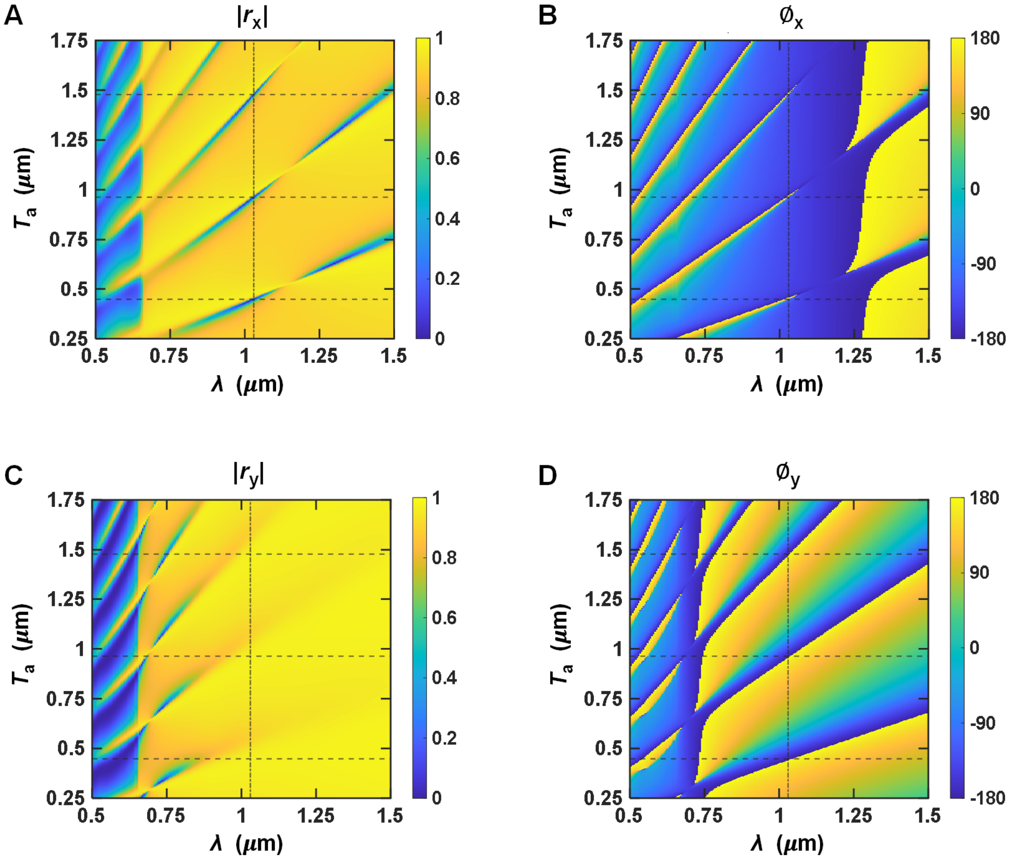

Fig. S2. Calculated complex reflection coefficients of the selected MEMS-OMS unit cell as a function of wavelength λ and air gap T_a. (**A**) Reflection amplitude $|r_x|$ and (**B**) phase ϕ_x under linearly *x*-polarized incident light. (**C**) Reflection amplitude $|r_y|$ and (**D**) phase ϕ_y under linearly *y*-polarized incident light. The vertical dashed line represents the operating wavelength, while the horizontal line represents the air gaps where the amplitudes significantly decrease for linearly *x*-polarized incident light in Fig. 2C. This structure is expected to exhibit perfect absorption conditions when the unit cell is above the minimum nanobrick oscillator strength (set by nanobrick dimension and density). Similarly, perfect absorption condition of *y*-polarized light occurs at the shorter wavelength.

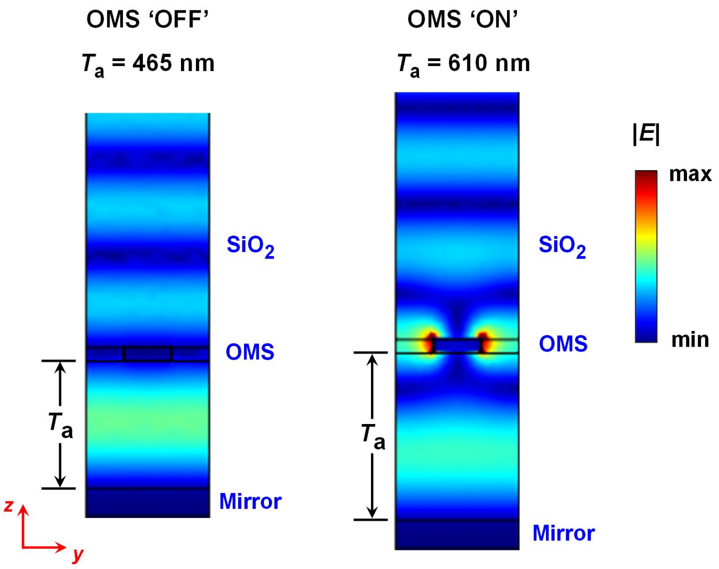

Fig. S3. Simulated normalized electric field norm (λ = 1030 nm) of the designed MEMS-OMS unit cell in the *y*-*z* plane under linearly polarized excitation, with the OMS layer at ‘OFF’ and ‘ON’ states, respectively.

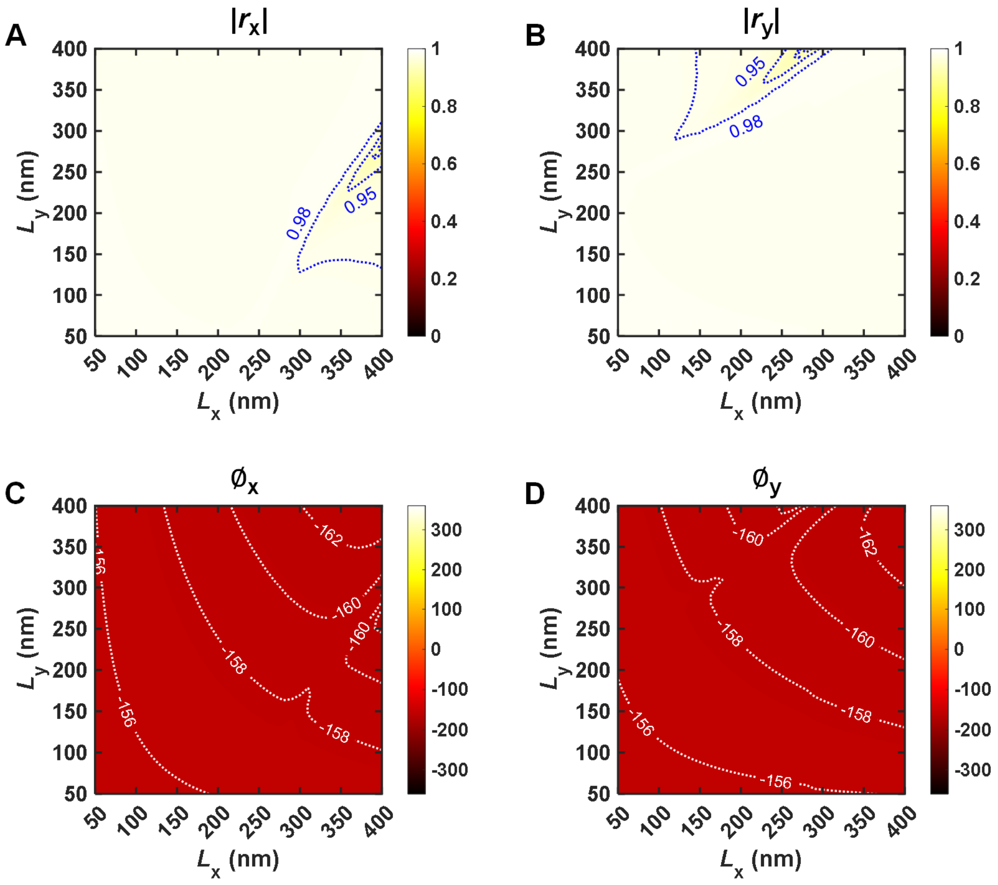

Fig. S4. Calculated complex reflection coefficients at mirror-like operation state (λ = 1030 nm). Reflection amplitude (**A, B**) $|r_{x(y)}|$ and (**C, D**) phase $\phi_{x(y)}$ under linearly *x*- and *y*-polarized incident light, respectively. At T_a = 465 nm, for all possible combinations of (L_x, L_y), the reflection amplitudes $|r_x|$ and $|r_y|$ are close to 1, and the phase difference $\Delta\phi_{xy}$ is close to 0°.

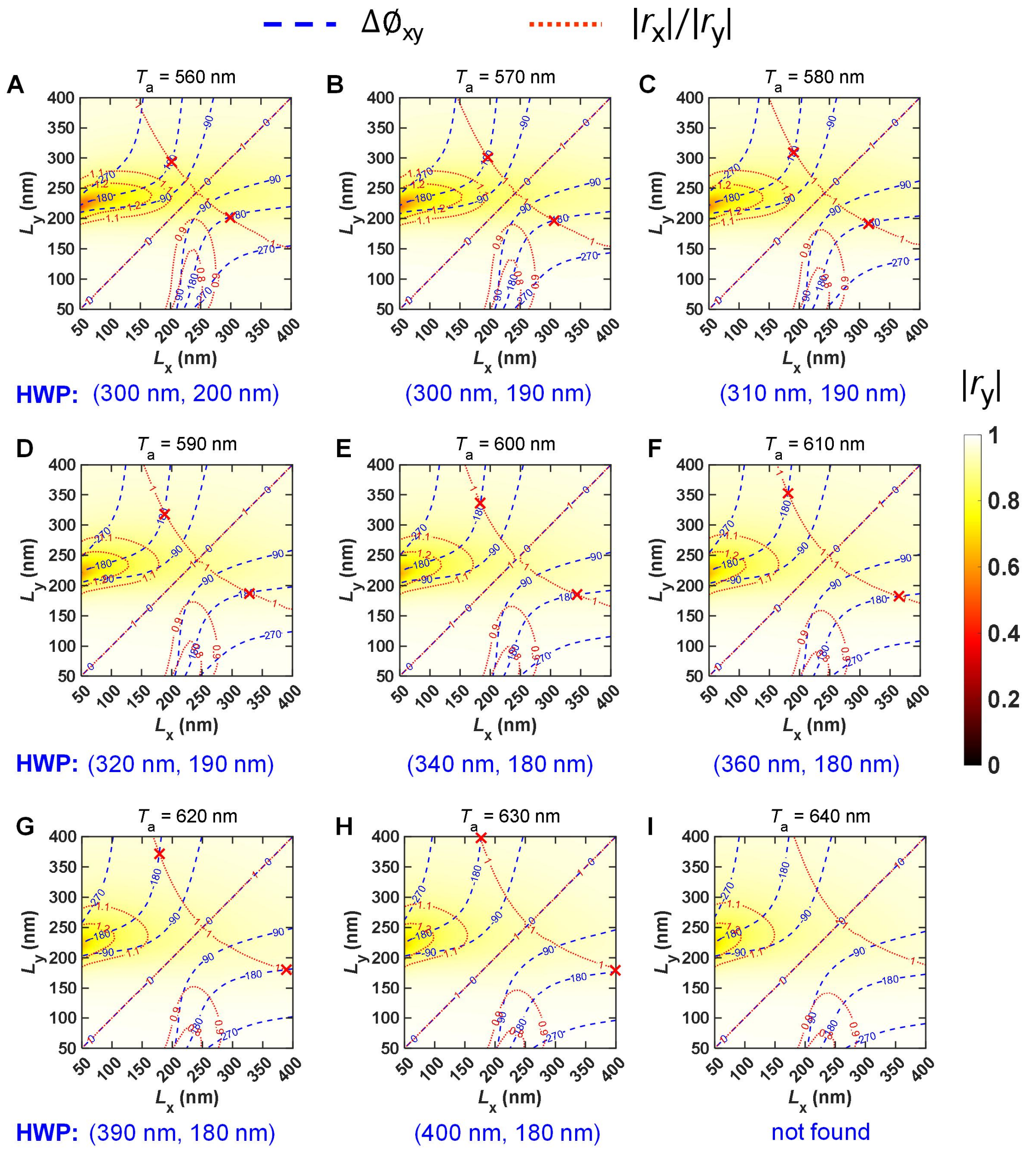

Fig. S5. Optimization of MEMS-OMS unit cell size for HWP functionality (λ = 1030 nm). (A-I) Calculated complex reflection coefficients of the MEMS-OMS for different sizes of nanobricks over different air gaps. Red cross markers represent the (L_x, L_y) combinations where the ratio of reflection amplitudes $|r_x|/|r_y|$ equals to 1 and the phase difference $\Delta\phi_{xy}$ equals to 180°.

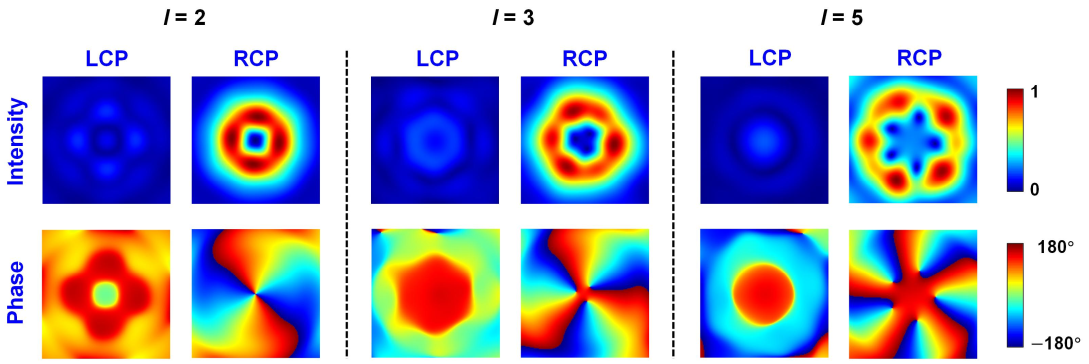

Fig. S6. Simulated intensity and phase distributions (λ = 1030 nm) in the reflected LCP and RCP channels from MEMS-OMSs (l = 2, 3, and 5), under RCP Gaussian beam incidence. Here, the vortex modes with l = 2, 3, and 5 dominate at T_a = 610 nm (k = 1), respectively.

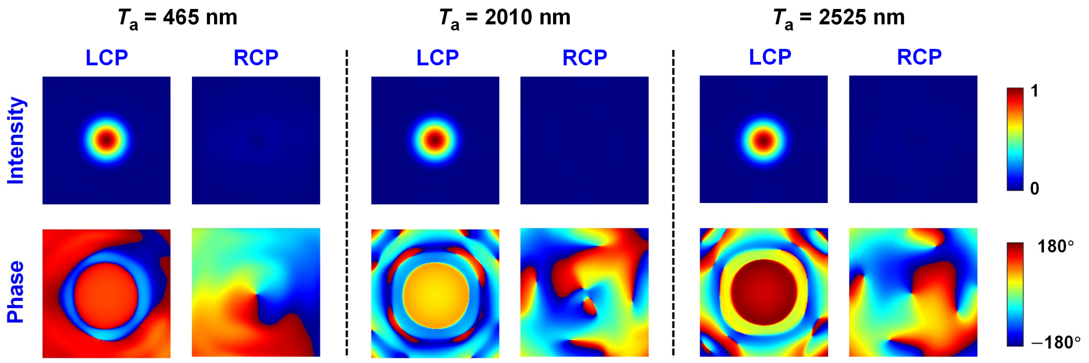

Fig. S7. Simulated intensity and phase distributions (λ = 1030 nm) in the reflected LCP and RCP channels from the MEMS-OMS (l = 1), under RCP Gaussian beam incidence. Here, the Gaussian mode (l = 0) dominates at T_a = 465, 2010, and 2525 nm (k = 1, 4, and 5), respectively.

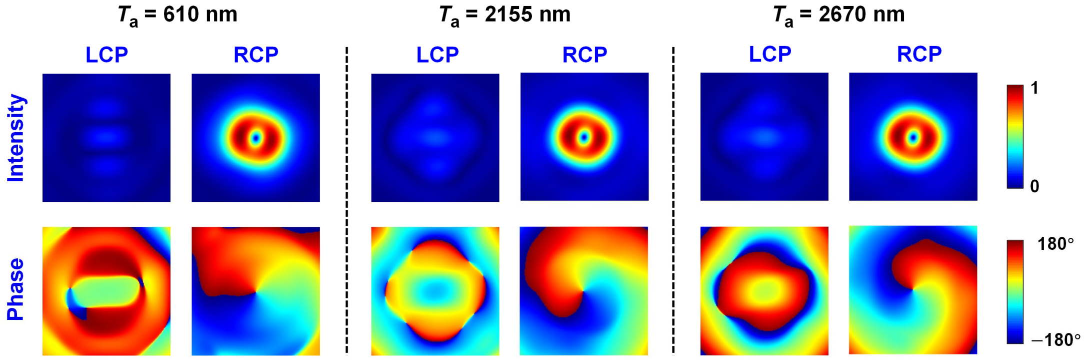

Fig. S8. Simulated intensity and phase distributions (λ = 1030 nm) in the reflected LCP and RCP channels from the MEMS-OMS (l = 1), under RCP Gaussian beam incidence. Here, the vortex mode (l = 1) dominates at T_a = 610, 2155, and 2670 nm (k = 1, 4, and 5), respectively.

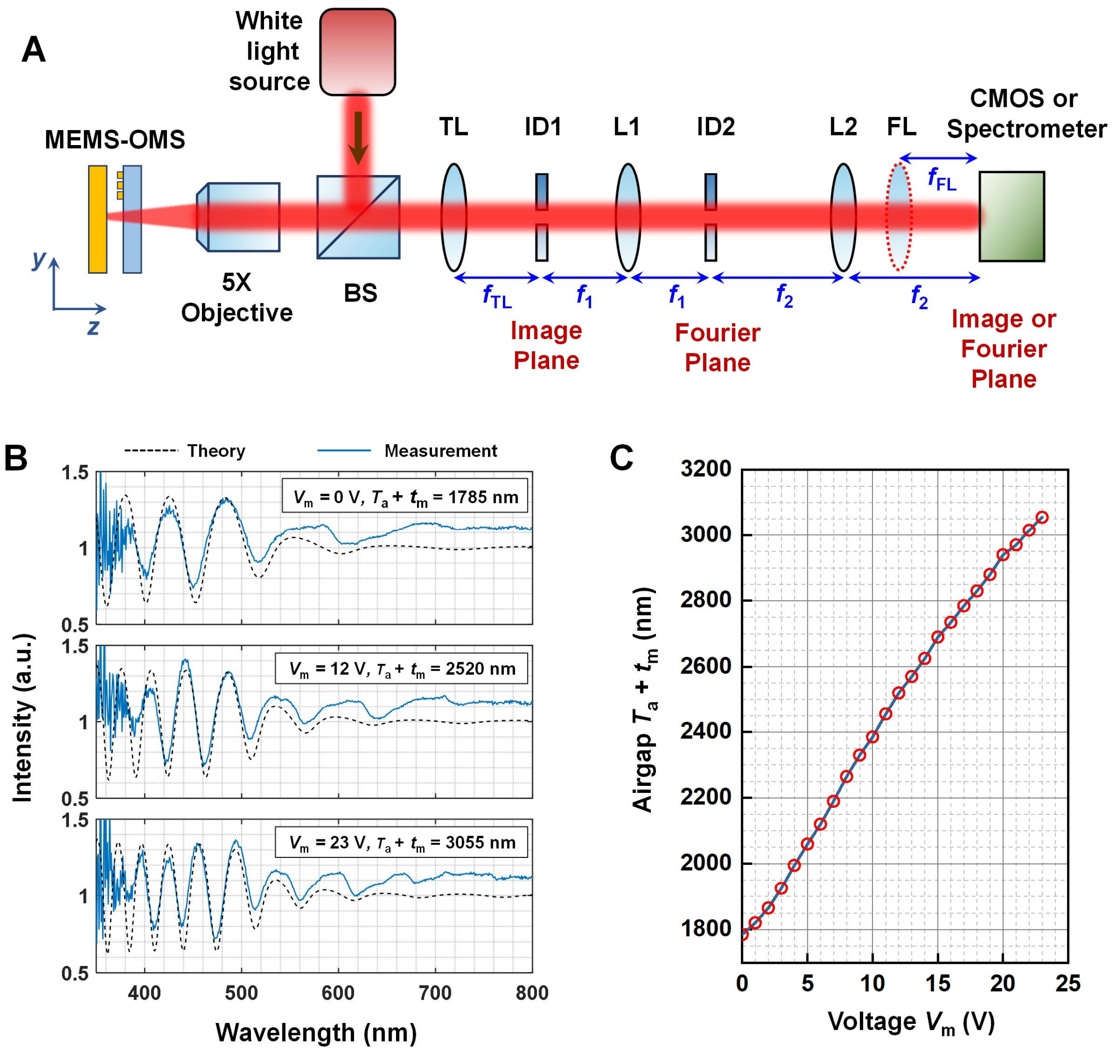

Fig. S9. Characterization of the air gap T_a as a function of the applied voltage V_m. (**A**) Experimental setup for measuring the MEMS-OMS separation. White light source: halogen lamp (wavelength range: 400 ~ 1000 nm); BS: Beam splitter; TL: Tube lens; ID1: Iris 1; L1: Lens 1 (f_1 = 150 mm); ID2: Iris 2; L2: Lens 2 (f_2 = 125 mm); FL: Flip lens (f_{FL} = 100 mm). (**B**) Typical reflection spectra from the non-structured area in MEMS-OMS, normalized by the reflected light from a reference 1-mm glass/100-nm gold structure. By fitting the measured normalized spectra with the theoretical spectra using the Fabry-Pérot etalon model, the separation T_a+ t_m between the MEMS mirror and the SiO_2 substrate can be estimated. (**C**) Estimated air gap (T_a + t_m) as a function of the applied voltage V_m. In experiment, the relationship between the applied voltage V_m and the air gap is slightly different each time (i.e., for mirror-like operation with T_a = 2010 nm, the applied voltage changes within a range of 4 ~ 4.5 V).

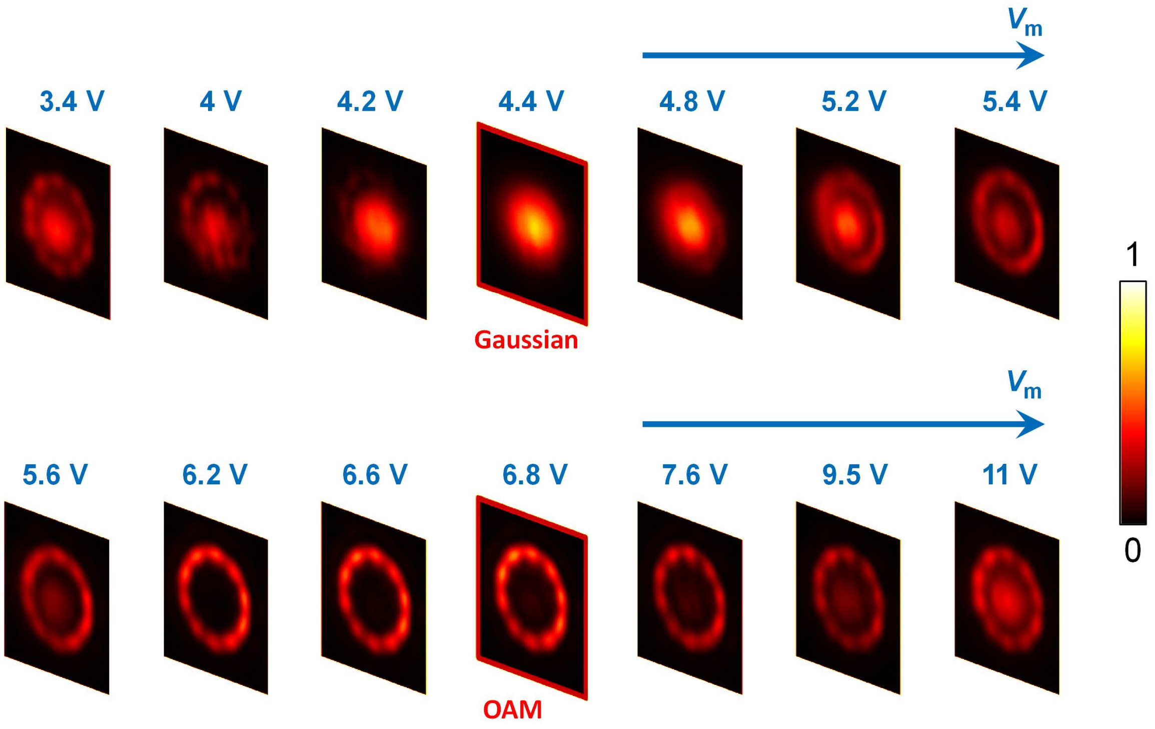

Fig. S10. Measured reflected beam profiles from the MEMS-OMS (l = 5) as a function of the applied voltage V_m, under CP Gaussian beam incidence (λ = 1030 nm). Note that the MEMS-OMS is not in the laser cavity.

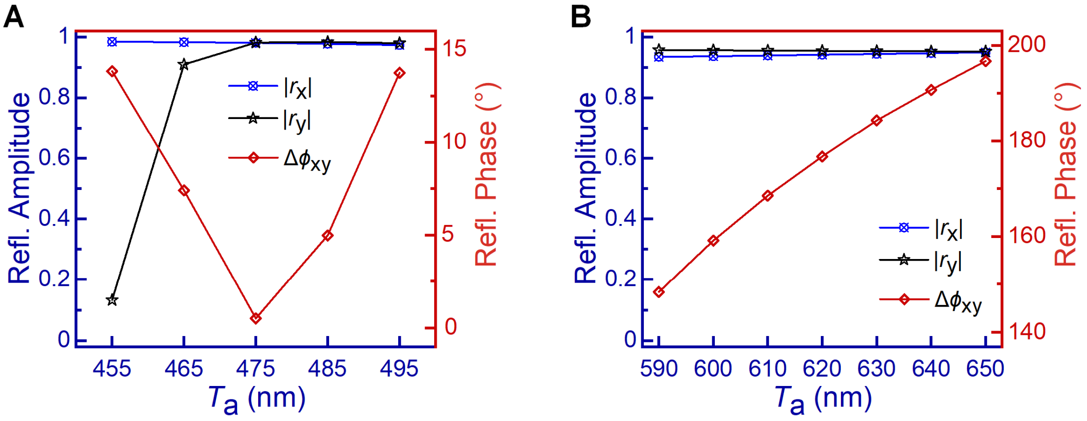

Fig. S11. Simulated reflection amplitudes $|r_{x(y)}|$ and phase difference $\Delta\phi_{xy}$ as a function of the air gap T_a for designed MEMS-OMS unit cell at 7° oblique incident angle (λ = 1030 nm). (**A**) mirror-like operation; (**B**) VWP operation.

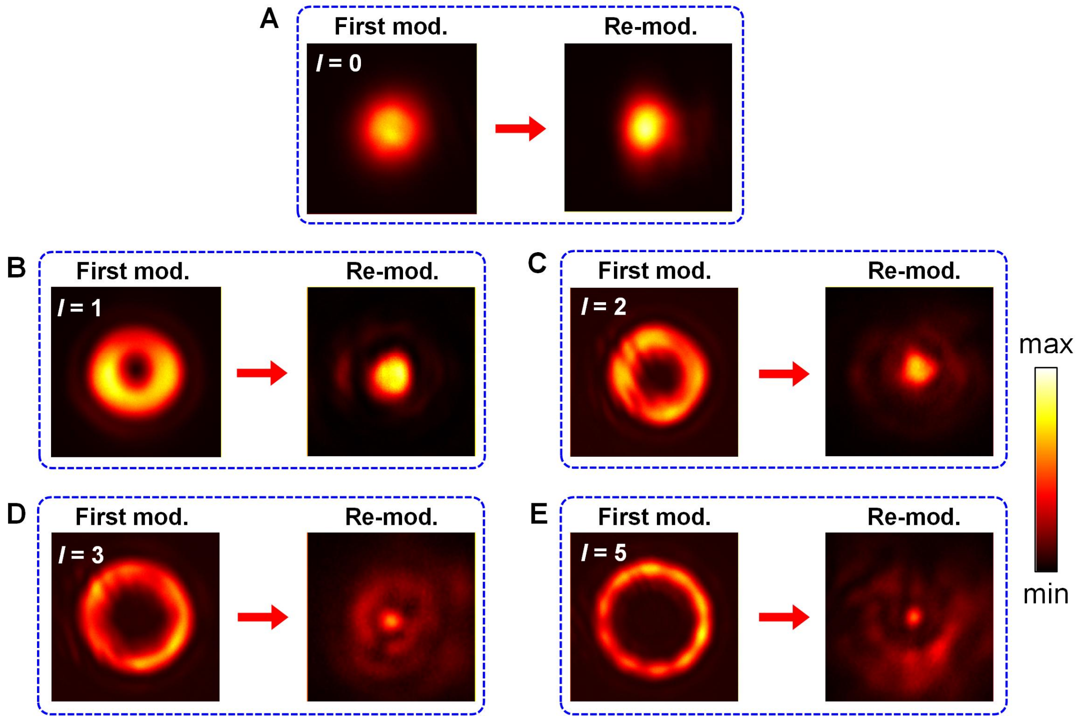

Fig. S12. Self-consistent verification of the beam profiles (λ = 1030 nm). Measured intensity distributions of the beams reflected by the MEMS-OMS once (i.e., first mod.) and twice (Re-mod.) for (**A**) Gaussian (l = 0) and (**B-E**) vortex (l = 1, 2, 3, and 5) modes.

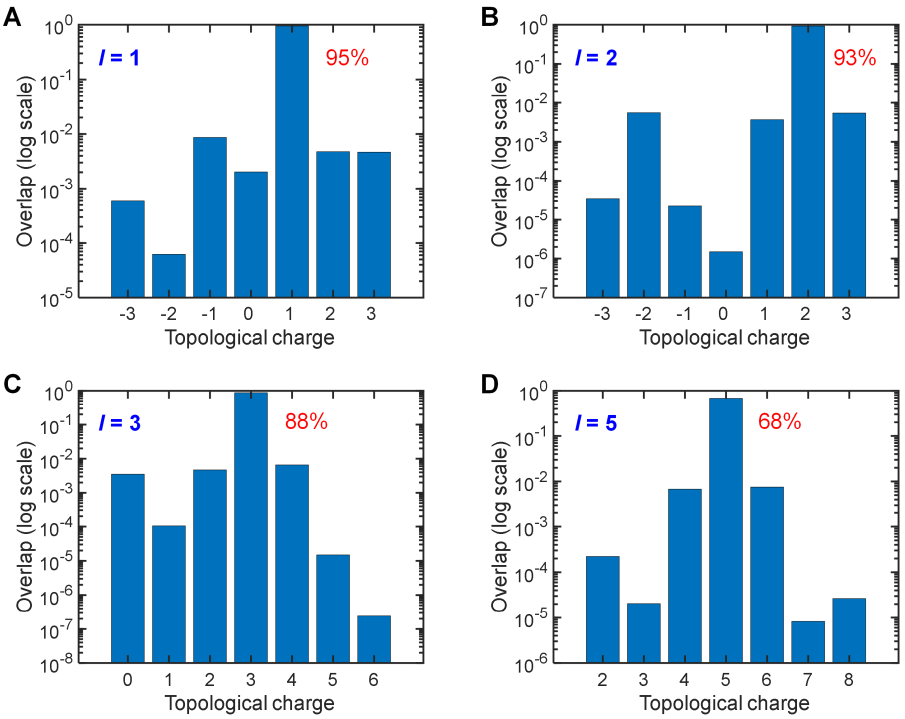

Fig. S13. Calculated modal decomposition of the simulated vortex beams (Fig. 3B). (**A-D**) Calculated mode weights of OAM modes with l = 1, 2, 3, and 5. OAM basis with radial mode index p = 0 can be expressed as: $\psi_{0,l}(r, \phi; w_0) \propto \left(\frac{r\sqrt{2}}{w_0}\right)^{|l|} \exp\left(-\frac{r^2}{w_0{}^2}\right) \exp(-il\phi)$, where w_0 is the beam waist, r is the radial distance from the beam axis, ϕ is azimuthal angle, and l is the topological charge. The subscripts 0 and l indicate the radial mode index and the azimuthal mode index. Note that the simulation is done with an overall metasurface size of 9 μm in diameter, whereas the actual metasurface fabricated in the experiment has a diameter of 100 μm.

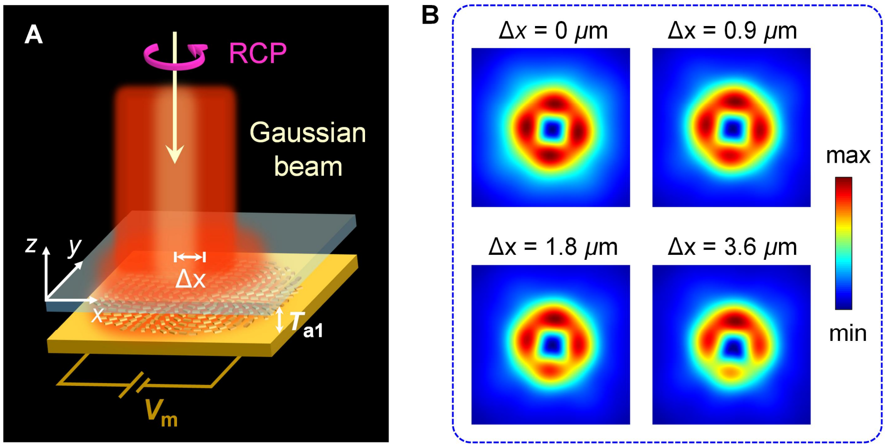

Fig. S14. Influence of the lateral misalignment between the incident beam and MEMS-OMS (l = 2). (**A**) Schematic of the lateral misalignment between the incident Gaussian beam and MEMS-OMS. Δx is the lateral misalignment of the incident beam along the *x*-direction. **(B)** Simulated intensity profiles of the generated vortex beams under incident Gaussian beams with varying Δx. Note that in this simulation the overall size of the MEMS-OMS is 14.4 μm in diameter, and the incident Gaussian beam waist is 9.6 μm.

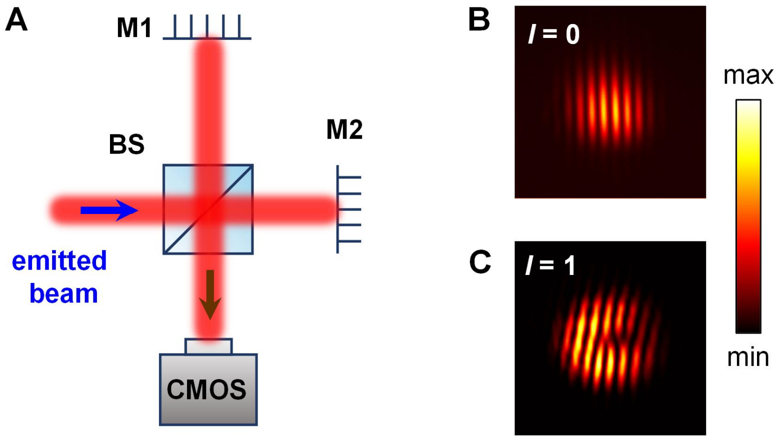

Fig. S15. Self-interference characterization. (**A**) Experimental setup of the Michelson interferometer for obtaining self-interference patterns. A beam splitter (BS) splits the emitted beam from laser cavity into two separate optical paths. The beams are then reflected by two mirrors (M1 and M2) respectively, interfere upon recombination at the beam splitter (BS), and are finally collected by a CMOS camera. (**B**) Typical self-interference pattern of a Gaussian mode. (**C**) Typical self-interference pattern of a l =1 vortex mode. The Gaussian beam exhibits distinct parallel interference fringes, attributable to its planar wavefront profile, while the vortex beam, characterized by a helical wavefront profile, features inverted fork-shaped fringes.

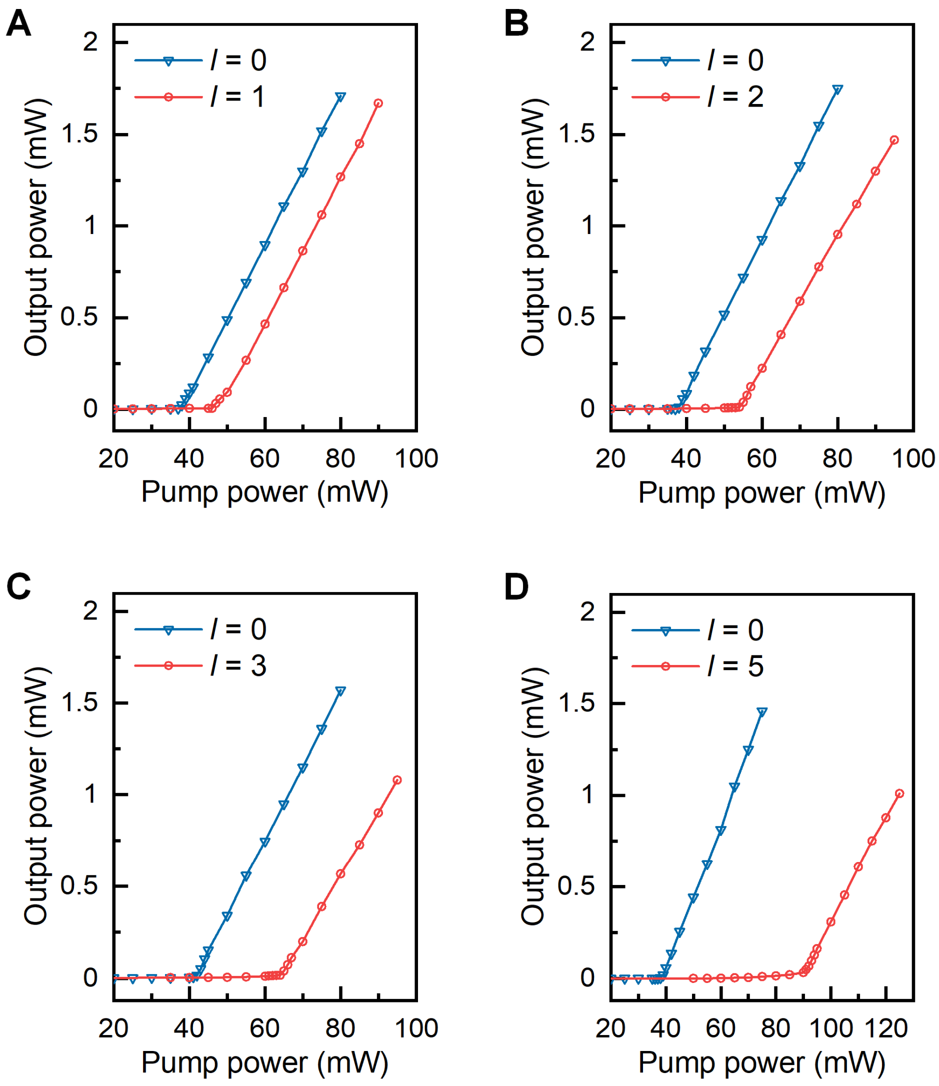

Fig. S16. Measured output power of the MEMS-OMS enabled mode-switchable lasers as a function of pump power. (**A-D**) Output characteristics of Gaussian (*l* = 0) and vortex (*l* = 1, 2, 3, and 5) operation states.

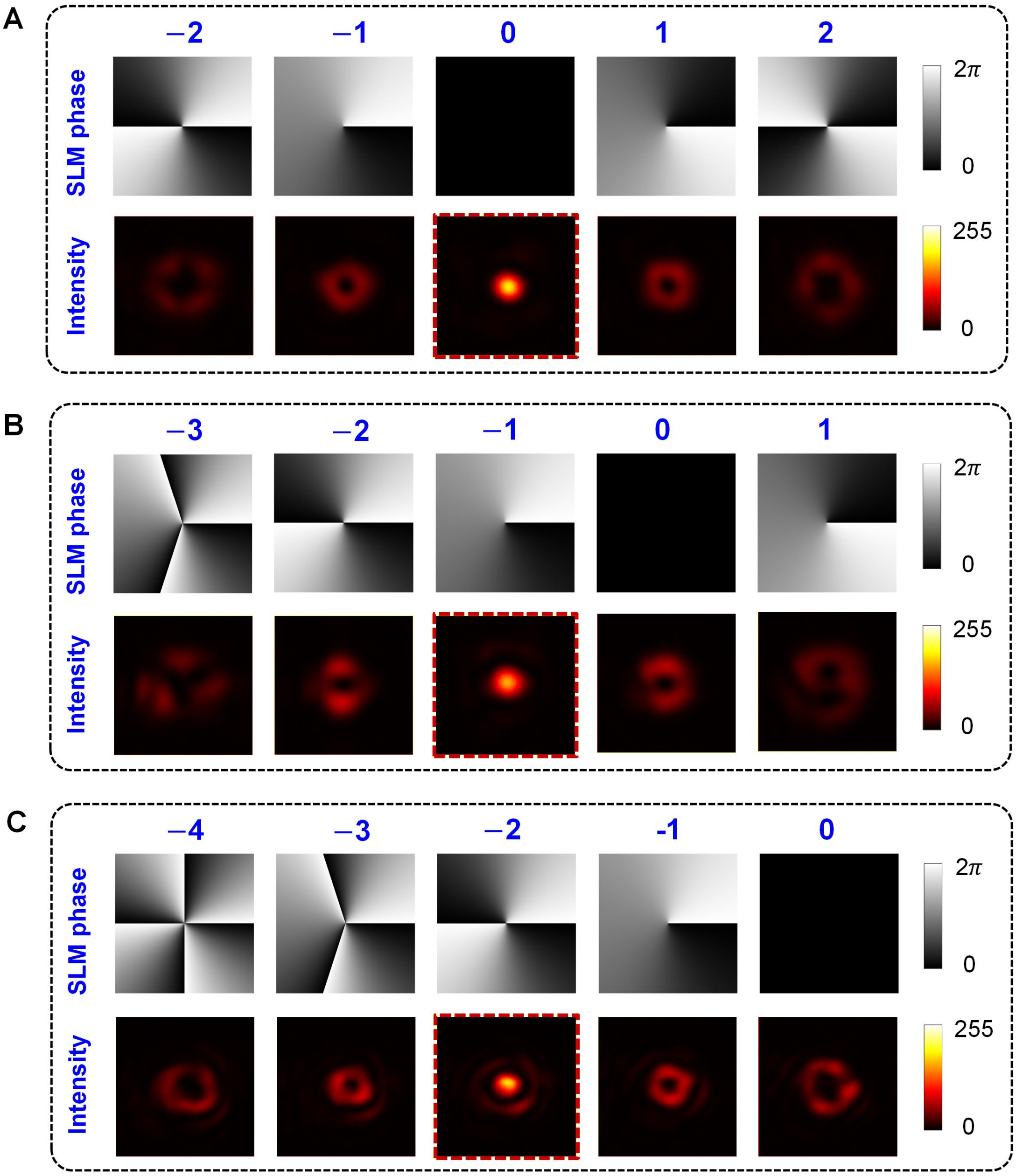

Fig. S17. OAM mode purity characterization. SLM phase profiles (first row) and intensity patterns modulated by SLM (second row) for analyzing emitted (**A**) Gaussian mode with $l = 0$, (**B**) vortex mode with $l = 1$, and (**C**) vortex mode with $l = 2$.

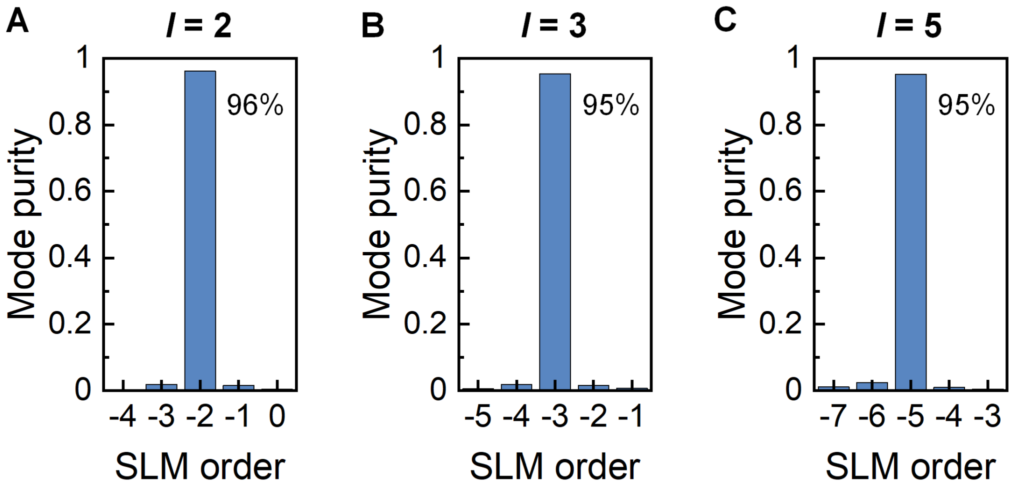

Fig. S18. Measured OAM mode purities of the beams emitted from the laser cavity. (**A-C**) Mode purity of the emitted vortex laser beams with topological charge of l = 2, 3, 5, respectively.

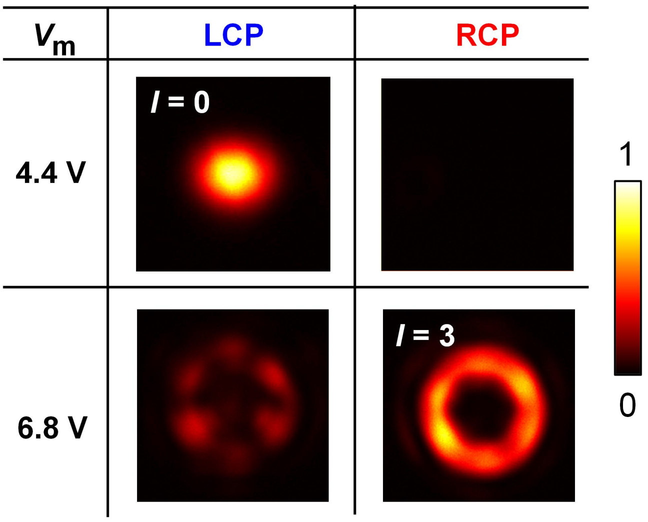

Fig. S19. Comparison of the emitted laser beam intensity at LCP/RCP channels with different MEMS-OMS actuation voltages (taking l = 3 MEMS-OMS as an example).

Movie S1. Extra-cavity mode switching.

Movie S2. Extra-cavity mode evolution.

Movie S3. Intra-cavity mode switching.
